# Analysis of Autophagy Genes in Microalgae: *Chlorella* as a Potential Model to Study Mechanism of Autophagy

**DOI:** 10.1371/journal.pone.0041826

**Published:** 2012-07-27

**Authors:** Qiao Jiang, Li Zhao, Junbiao Dai, Qingyu Wu

**Affiliations:** MOE Key Laboratory of Bioinformatics and Center for Epigenetics and Chromatin, School of Life Sciences, Tsinghua University, Beijing, China; University of Georgia, United States of America

## Abstract

**Background:**

Microalgae, with the ability to mitigate CO_2_ emission and produce carbohydrates and lipids, are considered one of the most promising resources for producing bioenergy. Recently, we discovered that autophagy plays a critical role in the metabolism of photosynthetic system and lipids production. So far, more than 30-autophagy related (ATG) genes in all subtypes of autophagy have been identified. However, compared with yeast and mammals, in silico and experimental research of autophagy pathways in microalgae remained limited and fragmentary.

**Principal Findings:**

In this article, we performed a genome-wide analysis of ATG genes in 7 microalgae species and explored their distributions, domain structures and evolution. Eighteen “core autophagy machinery” proteins, four mammalian-specific ATG proteins and more than 30 additional proteins (including “receptor-adaptor” complexes) in all subtypes of autophagy were analyzed. Data revealed that receptor proteins in cytoplasm-to-vacuole targeting and mitophagy seem to be absent in microalgae. However, most of the “core autophagy machinery” and mammalian-specific proteins are conserved among microalgae, except for the ATG9-cycling system in *Chlamydomonas reinhardtii* and the second ubiquitin-like protein conjugation complex in several algal species. The catalytic and binding residues in ATG3, ATG5, ATG7, ATG8, ATG10 and ATG12 are also conserved and the phylogenetic tree of ATG8 coincides well with the phylogenies. *Chlorella* contains the entire set of the core autophagy machinery. In addition, RT-PCR analysis verified that all crucial ATG genes tested are expressed during autophagy in both *Chlorella* and *Chlamydomonas reinhardtii*. Finally, we discovered that addition of 3-Methyladenine (a PI3K specific inhibitor) could suppress the formation of autophagic vacuoles in *Chlorella*.

**Conclusions:**

Taken together, *Chlorella* may represent a potential model organism to investigate autophagy pathways in photosynthetic eukaryotes. The study will not only promote understanding of the general features of autophagic pathways, but also benefit the production of *Chlorella*-derived biofuel with future commercial applications.

## Introduction

Microalgae represent a group of tiny and usually unicellular algae, including numerous marine and freshwater species [1]. Microalgae have long been considered potential resources for renewable bioenergy production [2]–[4]. The cellular lipids in several species of microalgae can be used to produce biofuel. For instance, *Chlorella protothecoides* is one of the promising model oleaginous microalgae to produce commercially viable biodiesel because of its rapid biomass production, high photosynthetic efficiency and high lipid content (50.3% of dry cell weight) [5]–[8]. Algae use light energy to convert water and carbon dioxide into glucose and oxygen. Ideally, microalgal biodiesel is carbon-neutral and feasible without affecting global food supply [1]. Algal biofuel research has been a hot topic and attracted experts both in biotechnology and biochemical engineering. However, most of these studies focused on algal cultivation and transesterification. Molecular and cellular mechanisms concerning lipid accumulation in model oleaginous microalgae remains elusive. Recently, we identified that autophagy plays a critical role in the metabolism of photosynthetical system and in the production of lipids in *Chlorella*. Inhibition of autophagy impairs the breakdown of chloroplasts and decreases lipid accumulation (Jiang Q. et. al., manuscript in preparation). Thus, a better understanding of the cellular and molecular aspects of autophagy is required for the production of biofuel and mitigating CO_2_ emission.

Programmed cell death (PCD) is a crucial mechanism involved in many physiological and pathological processes. Studies of PCD used to focus on apoptosis (Type I PCD, self-killing) and recently, autophagy (Type II PCD, self-eating) became an active field of study. Autophagy is a conserved catabolic process for the degradation of long-lived organelles and proteins, which consists of several sequential processes. The early stage of autophagy includes induction and formation of autophagosomes and the late stage includes fusion of autophagosome and lysosome/vacuole, degradation and lysosome/vacuole reformation [9]. In metazoa, autophagic dysfunction is implicated in cancer, ageing, and neurodegeneration [10], [11]. In plants, autophagy induced by starvation or senescence is responsible for nutrient recycling and negative regulation of apoptosis, and has a pro-death function in hypersensitive cell death [12], [13]. Autophagy studies in filamentous fungi *Podospora anserina*, *Aspergillus oryzae* and *A. fumigatus* suggested the role of autophagy in fungal differentiation [14], [15].

Various forms of autophagy, either selective or not, have been discovered, including macroautophagy, microautophagy, Cvt (cytoplasm-to-vacuole targeting), and pexophagy. Macroautophagy is a mechanism responsible for the degradation of cytoplasm using specialized cytosolic vesicles, while Cvt is a selective pathway in which at least two precursor hydrolases are transported to the vacuole [16]. Pexophagy, the selective autophagic degradation of peroxisomes, has been widely studied in *Pichia pastoris* [17]. Certain organelles can also be selectively degraded through autophagy, including mitochondria (mitophagy) and ribosomes (ribophagy) [18], [19].

ATG (**A**u**t**opha**g**y-related) genes encode proteins required for autophagy and were first identified in *S. cerevisiae*. Afterwards, several putative ATG genes maintaining sequence and functional conservation were revealed in yeast, filamentous fungi, mammalian, and plant genomes [20], [21]. Besides, **TOR** (the target of rapamycin) is a conserved Ser/Thr kinase that regulates autophagy from yeast to plants and mammals [22]. In addition, several proteins absent in yeast are crucial for autophagy in mammals, including UVRAG (UV irradiation resistance-associated gene, for activating VPS34), DRAM (Damage-Regulated Autophagy Modulator, like TOR), FIP200 (FAK family-interacting protein of 200 kDa) and ATG101 (mediating mTOR signalling) [23]–[27]. To date, more than thirty ATG proteins have been identified in various autophagy pathways. ATG genes required for autophagosome formation are termed the “core autophagy machinery”. Seventeen out of 34 known ATG proteins constitute three series of core autophagic machinery, including the **ATG9-Cycling system** (transmembrane protein ATG9, the ATG1 kinase complex ATG1, ATG13, ATG2, ATG18, ATG23 and ATG27), the **phosphatidylinositol 3-kinase (PI3K) complex** (VPS34, ATG6/VPS30, ATG14, vacuolar protein sorting VPS15, and VPS38), and the **ubiquitin-like protein (Ubl) conjugation system** (Ubl protein ATG8, ATG12, ATG3, ATG10, ATG7, ATG4, ATG5 and ATG16) [28].

Experimental evidence for the presence of autophagic-like vacuoles in microalgae has been described in four species, including *C. reinhardtii* treated with rapamycin or a mutant *C. reinhardtii* strain lacking phytoene synthase, diatom *Cyclotella meneghiniana* exposed to chlorinated benzenes, and uninfluenced growth of *Dunaliella primolecta* [29]–[32]. Putative ATG proteins have been previously identified in three algal species [33]. The presence and function of TOR genes have also been surveyed in microalgae, which demonstrated the presence of TOR homolog in *Chlamydomonas reinhardtii*. After treatment with rapamycin, FKBP12 (the 12-kD FK506-binding protein) binds rapamycin and inactivates TOR. Besides, in silico analysis of TOR was performed and several putative homologs of TOR were identified as well [32], [33]. However, experimental evidence and in-silico studies of autophagy pathways in model oleaginous microalgae have not been reported.

In order to identify ATG genes and investigate autophagic pathway in microalgae, including promising oleaginous microalgae, we manually checked all of the ATG genes and explored their distributions, domain structures and evolution. More than 40 related proteins in the core machinery and representative subtypes were used as queries. The results showed that more ATG genes are present than currently annotated in databases, because certain genes were either missed or incorrectly annotated. Furthermore, RT-PCR was performed for several identified ATG genes to confirm the existence of specific mRNAs in *Chlorella* and *Chlamydomonas*. The results suggest that *Chlorella*, one of the model oleaginous microalgae, could become a potential model organism to investigate autophagy pathways in photosynthetic eukaryotes. The study will not only promote understanding of the general features of autophagic pathways, but also benefit the production of *Chlorella*-derived biofuel for future commercial applications.

## Results

### Identification of ATG genes in microalgae and choanoflagellate

Because the molecular insights of the autophagic pathway mainly come from yeast species, most of the ATG proteins are identified in *S. cerevisiae* (Sc) and *P. pastoris* (Pp). Putative homologs of the Sc ATG or Pp ATG proteins were identified in 9 microalgae genomes (Table 1). For comparison, we also searched the genome of the marine choanoflagellate *Monosiga brevicollis*, which is the closest unicellular relative of animals. However, we cannot exclude the possibility that, in certain species, not all of the ATG sequences could be identified because of excessively high evolutional divergence.

**Table 1:** Species analyzed in this study.

Abbreviation	Organisms	Key Feature	Environment	Prior experimental evidence of autophagy
Cr	*Chlamydomonas reinhardtii*	Chlorophyceae (green alga, Model organism)	Freshwater	Yes
Cv	*Chlorella* sp. NC64A (*Chlorella variabilis*)	Trebouxiophyceae (green alga)	Freshwater; symbiotic	Unpublished results
Mb	*Monosiga brevicollis*	Choanoflagellate (protist, closest animal relative)	Marine; heterotrophic	NA
Mp	*Micromonas pusilla* CCMP1545	Prasinophyceae (ancient green alga)	Marine (temperate coastal waters)	NA
Mr	*Micromonas pusilla* NOUM17 (*Micromonas* sp. RCC299)	Prasinophyceae (ancient green alga)	Marine (tropical waters)	NA
Ol	*Ostreococcus lucimarinus*	Prasinophyceae (ancient green alga)	Marine (upper water column)	NA
Ot	*Ostreococcus tauri*	Prasinophyceae (ancient green alga)	Marine (upper water column)	NA
Pt	*Phaeodactylum tricornutum* CCAP1055/1	Diatom	Marine	NA
Tp	*Thalassiosira pseudonana* CCMP1335	Diatom	Marine	NA
Vc	*Volvox carteri*	Chlorophyceae (green alga)	Freshwater	NA

Na, not present.

### ATG proteins involved in the core machinery

The core autophagy machinery in yeast and plants can be divided into three systems: 1) ATG9-Cycling, 2) the phosphatidylinositol 3-kinase (PI3K), and 3) the ubiquitin-like protein complex [21]. Most genes belonging to the core machinery have been identified in microalgae and choanoflagellates (summarized in Table 2–9, domain structure included).

**Table 2:** Distribution of ATG proteins and domains in microalgae and *M. brevicollis*: ATG proteins involved in ATG9-Cycling system.

	ATG1 (NP_011335.1)	ATG2 (NP_014157.1)	ATG9 (NP_010132.1)	ATG13 (NP_015511.1)	ATG18 (NP_444297.1)	ATG23 (NP_013535.1)	ATG27 (NP_012357.2)
	(PKc_like+DUF3543+S_TKc)	(ATG_C)	(APG9)	(ATG13)	(WD40)	(no)	(ATG27)
*Chlorella* sp. NC64A (*Chlorella variabilis*)	EFN52208.1(PKc_like+S_TKc) EFN56161.1(S_TKc+STKc_AGC)	Na	EFN53041.1 (APG9)	EFN59140.1 (ATG13)	EFN51454.1(WD40)EFN58088.1(WD40)	Na	Na
*Chlamydomonas reinhardtii*	XP_001695074.1(PKc_like+S_TKc) XP_001696712.1(PKc_like+Pkinase)	Na	Na	Na	XP_001698567.1(WD40) XP_001689953.1(WD40)	Na	Na
*Micromonas pusilla* CCMP1545	XP_003059119.1(PKc_like+S_TKc)	XP_003060662.1 (ATG_C)	XP_003057426.1 (APG9)	XP_003061623.1 (ATG13)	XP_003059105.1(WD40) XP_003060952.1(WD40)	Na	Na
*Micromonas sp.* RCC299	XP_002500392.1(PKc_like+S_TKc)	XP_002504952.1 (ATG_C+MRS6)	XP_002502107.1 (APG9)	XP_002509135.1 (ATG13)	XP_002500515.1(WD40+RING+Prp19) XP_002500377.1(WD40) XP_002955876.1(WD40) XP_002503507.1(WD40)	Na	Na
*Monosiga brevicollis*	XP_001749266.1(PKc_like+S_TKc)	XP_001743636.1 (ATG_C)	XP_001749415.1 (APG9)	Na	XP_001743556.1(WD40) XP_001746830.1(WD40) XP_001749074.1(WD40) XP_001746866.1(WD40+SMC_prok_A)	Na	XP_001748990.1(ATG27) XP_001748596.1(ATG27) XP_001748431.1(ATG27+Frag1+WSC+Somatomedin_B+Kelch_1)
*Ostreococcus lucimarinus*	XP_001418427.1(PKc_like+S_TKc) XP_001420104.1(S_TKc+STKc_AGC)	XP_001417521.1 (ATG_C)	XP_001418016.1 (APG9)	XP_001420417.1 (ATG13)	XP_001418375.1(WD40) XP_001421096.1(WD40)	Na	Na
*Ostreococcus tauri*	XP_003079793.1(PKc_like) XP_003081539.1(S_TKc+STKc_AGC)	XP_003078813.1 (ATG_C)	XP_003079378.1 (APG9)	XP_003081852.1 (ATG13)	XP_003079686.1(WD40+COG4886) XP_003079697.1(WD40+AdoMet_MTase)	Na	Na
*Phaeodactylum tricornutum*	XP_002178626.1(PKc_like+S_TKc) XP_002180851.1(PKc_like+STYKc)	XP_002185265.1 (ATG_C) XP_002184167.1 (ATG_C)	XP_002180897.1 (APG9)	XP_002176466.1 (ATG13)	XP_002182364.1(WD40+PRP4) XP_002182356.1(WD40)	Na	Na
*Thalassiosira pseudonana*	XP_002291487.1(PKc_like+S_TKc) XP_002290245.1(S_TKc+STKc_AGC)	Na	XP_002288788.1 (APG9)	XP_002294456.1 (ATG13)	XP_002292898.1 (WD40)	Na	Na
*Volvox carteri*	XP_002948563.1(PKc_like+S_TKc) XP_002956722.1(PKc_like+Pkinase)	XP_002951181.1 (ATG_C+MRS6+DUF946)	XP_002952621.1 (APG9)	XP_002954490.1 (ATG13)	XP_002954593.1(WD40+ PKD_channel+COG2319+Ion_trans) XP_002955876.1(WD40) XP_002947339.1(WD40+DNA_BRE_C)	Na	Na

Na, not present or not identifiable; numbers indicate GenBank DNA accession numbers.

**Table 3:** Distribution of ATG proteins and domains in microalgae and *M. brevicollis*: ATG proteins involved in PI3K complex.

	ATG6 (NP_015205.1) (APG6)	ATG14 (NP_009686.1) (ATG14)	VPS34 (NP_013341.1) (PI3Kc_III+PI3Ka_III+C2_PI3K_class_III)	VPS15 (NP_009655.2) (Pkinase+WD40)
*Chlorella* sp. NC64A (*Chlorella variabilis*)	CP04629(APG6)	EFN52878(ATG14+NIF) EFN51481(ATG14)	EFN50586.1(C2) EFN55063.1(PI3Kc_like+FATC) EFN54838.1(PI3Kc_like+PI3Ka)	EFN54741.1(Pkinase+WD40+HEAT) ACQ83472.1(Pkinase+NAF+DUF552) EFN59818.1(Pkinase)
*Chlamydomonas reinhardtii*	XP_001689505.1(APG6)	XP_001693556(ATG14)	AAC50017.1(PI3Kc_III+PI3Ka_III+PI3Kc_like) XP_001700926.1(PI4Kc_III_alpha+PI3Ka)XP_001689631.1 (PI3Kc_III+PI3Ka_III+C2+PI3Kc_like)	XP_001703148.1(Pkinase) XP_001703541.1(Pkinase+IQ)
*Micromonas pusilla* CCMP1545	XP_003060474.1(APG6)	XP_003064431(ATG14)	XP_003062450.1(PI3Kc_III+PI3Ka_III+C2)	XP_003055278.1 (Pkinase+WD40+HEAT)
*Micromonas sp.* RCC299	XP_002505141.1(APG6)	XP_002502757 (ATG14+BAR)	XP_002506109.1 (PI3Kc_III+PI3Ka_III+C2)	XP_002499874.1(Pkinase)
*Monosiga brevicollis*	XP_001748523.1 (APG6+RPN7+PCI)	XP_001745375 (ATG14+FmrO+GAS)	XP_001748498.1(PI3Kc+C2A_PI3K_class_II+PI3Ka+C2A_SLP+PX_domain+SH3) XP_001744392.1(PI3Kc_III) XP_001744858.1 (PI3Kc+PI3Ka+C2+PI3K_rbd+PI3K_p85B)	XP_001743460.1(Pkinase) XP_001749069.1(Pkinase) XP_001750681.1(Pkinase) XP_001749118.1 (Pkinase+WD40+2_5_RNA_ligase)
*Ostreococcus lucimarinus*	XP_001417602.1(APG6)	XP_001422247.1(ATG14)XP_001421563.1(ATG14)	XP_001416907.1(PI3Kc_III+PI3Ka_III)	XP_001417017.1(Pkinase+WD40+HEAT)
*Ostreococcus tauri*	XP_003079004.1(APG6)	XP_003084186(ATG14) XP_003083512(ATG14)	XP_003078099.1(PI3Kc_III+PI3Ka_III+C2)	XP_003078318.1(Pkinase+WD40+HEAT)
*Phaeodactylum tricornutum*	XP_002176511.1(APG6) XP_002177919.1(APG6)	XP_002177421 (ATG14) XP_002176147 (ATG14) XP_002185098(ATG14)	XP_002184171.1(PI3Kc_like)	XP_002184017.1(Pkinase+WD40) XP_002178422.1 (Pkinase+TPR_2+TPR_1)
*Thalassiosira pseudonana*	XP_002292845.1(APG6) XP_002291400.1(APG6)	XP_002294494.1(ATG14) XP_002288406(ATG14)	XP_002293246.1(PI3Kc_like+PI3Ka_III) XP_002296868.1(PI3Kc_like)	XP_002288602.1(Pkinase) XP_002292529.1(Pkinase+DUF2271) XP_002293876.1(Pkinase) XP_002289061.1(Pkinase+WD40+IKI3+MMS19_N) XP_002288266.1(Pkinase+MCR_beta+Phage_head_chap)
*Volvox carteri*	XP_002952061.1(APG6)	XP_002948890(ATG14) XP_002953012(ATG14)	XP_002954001.1 (PI3Kc_III+PI3Ka_III+PI3Kc_like)	XP_002947818.1(Pkinase+WD40+RRM_1+Poxvirus_B22R+Otopetrin) XP_002951746.1(Pkinase +IQ) XP_002959907.1(Pkinase+Pex24p) XP_002950395.1(Pkinase) XP_002951452.1(Pkinase)

Na, not present or not identifiable; numbers indicate GenBank DNA accession numbers.

**Table 4:** Distribution of ATG proteins and domains in microalgae and *M. brevicollis*: ATG proteins involved in Ubiquitin-Like Protein Conjugation Systems.

	ATG3 (NP_014404.1) (Autophagy_N+Autophagy_act_C+Autophagy_Cterm)	ATG4 (NP_014176.2) (Peptidase_C54)	ATG5 (NP_015176.1) (APG5)	ATG7 (NP_012041.1) (Apg7+E1_like_apg7)	ATG8 (NP_009475.1) (GABARAP)/(XP_001699190.1) (GABARAP)	ATG10 (NP_013058.1) (Autophagy_act_C)	ATG12 (NP_009776.1) (APG12)	ATG16 (NP_013882.1) (APG16)
*Chlorella* sp. NC64A (*Chlorella variabilis*)	EFN54110.1 (Autophagy_N+Autophagy_act_C+Autophagy_Cterm) EFN54044.1(Autophagy_act_C)	EFN56996.1 (Peptidase_C54) EFN56995.1 (Peptidase_C54)	EFN59373.1 (APG5)	EFN52000.1(E1_like_apg7+E1_enzyme_family)	EFN52105.1 (GABARAP)	EFN54044.1 (Autophagy_act_C)	EFN51330.1 (APG12)	Na
*Chlamydomonas reinhardtii*	XP_001699795.1 (Autophagy_Cterm+Autophagy_act_C+Autophagy_N)	XP_001691049.1 (Peptidase_C54)	XP_001692662.1 (APG5)	XP_001703365.1(E1_like_apg7+E1_enzyme_family)	XP_001699190.1 (GABARAP)	XP_001692913.1 (Autophagy_act_C)	XP_001702830.1 (APG12)	Na
*Micromonas pusilla* CCMP1545	XP_003062013.1 (Autophagy_Cterm+Autophagy_act_C+Autophagy_N)	Na	XP_003064603.1 (APG5)	XP_003060175.1(E1_like_apg7+E1_enzyme_family)	XP_003063071.1 (GABARAP)	Na	XP_003058399.1 (APG12)	Na
*Micromonas sp.* RCC299	XP_002508119.1 (Autophagy_Cterm+Autophagy_act_C+Autophagy_N)	XP_002504432.1 (Peptidase_C54)	Na	XP_002504186.1(E1_like_apg7+E1_enzyme_family)	XP_002506884.1 (GABARAP)	Na	XP_002503341.1 (APG12)	XP_002502794.1(ATG16+PGAM+WD40)
*Monosiga brevicollis*	XP_001748852.1 (Autophagy_Cterm+Autophagy_N+Autophagy_act_C)	XP_001745089.1 (Peptidase_C54) XP_001747503.1 (Peptidase_C54)	XP_001747184.1 (APG5+WHEP-TRS+tRNA-synt_2b+HGTP_anticodon)	Na	XP_001748106.1 (GABARAP)	Na	Na	XP_001749313.1(ATG16+WD40)
*Ostreococcus lucimarinus*	XP_001418543.1 (Autophagy_Cterm+Autophagy_act_C+Autophagy_N)	XP_001417407.1 (Peptidase_C54)	XP_001422251.1 (APG5)	XP_001418514.1 (Apg7+E1_like_apg7)	XP_001415651.1 (GABARAP)	Na	XP_001418371.1 (APG12)	XP_001420801.1(ATG16+WD40)
*Ostreococcus tauri*	XP_003080019.1 (Autophagy_Cterm+Autophagy_act_C)	XP_003078525.1 (Peptidase_C54)	XP_003084193.1 (APG5)	XP_003079963.1 (Apg7)	XP_003074617.1 (GABARAP)	Na	XP_003079693.1 (APG12+GST_C)	Na
*Phaeodactylum tricornutum*	XP_002176659.1 (Autophagy_Cterm+Autophagy_act_C+Autophagy_N)	XP_002185127.1 (Peptidase_C54)	XP_002186339.1 (APG5)	Na	XP_002185239.1 (GABARAP)	Na	Na	XP_002177918.1(ATG16+DUF745+WD40)
*Thalassiosira pseudonana*	XP_002295882.1 (Autophagy_Cterm+Autophagy_act_C+Autophagy_N)	XP_002294341.1 (Peptidase_C54)	Na	XP_002289307.1 (E1_like_apg7+E1_enzyme_family)	XP_002287173.1 (GABARAP)	Na	XP_002291258.1 (APG12)	XP_002289194.1(ATG16+WD40)
*Volvox carteri*	XP_002952517.1 (Autophagy_Cterm+Autophagy_act_C+Autophagy_N)	XP_002948308.1 (Peptidase_C54)	Na	XP_002951753.1 (E1_like_apg7+E1_enzyme_family)	XP_002952089.1 (GABARAP)	Na	Na	XP_002948303.1(ATG16+BicD+IncA+HAP1_N)

Na, not present or not identifiable; numbers indicate GenBank DNA accession numbers.

**Table 5:** Distribution of ATG proteins and domains in microalgae and *M. brevicollis*: ATG proteins exclusively involved in non-selective autophagy (including macroautophagy and microautophagy).

	ATG17 (NP_013527.1) (APG17)	ATG28 (XP_002491513.1) (Na)	ATG29 (NP_015159.1) (Na)/(ABO31324.1)(Na)	ATG31 (NP_010305.1) (ATG31)
*Chlorella* sp. NC64A (*Chlorella variabilis*)	EFN58270.1(APG17+ubiquitin+DELLA)	Na	Na	Na
*Chlamydomonas reinhardtii*	Na	Na	Na	Na
*Micromonas pusilla* CCMP1545	XP_003062461.1(APG17+Nucleoplasmin+TMF_DNA_bd)	Na	Na	Na
*Micromonas sp.* RCC299	XP_002506097.1(APG17+ubiquitin)	Na	Na	Na
*Monosiga brevicollis*	Na	Na	Na	Na
*Ostreococcus lucimarinus*	Na	Na	Na	Na
*Ostreococcus tauri*	Na	Na	Na	Na
*Phaeodactylum tricornutum*	XP_002186517.1(APG17+ATG11)	Na	Na	Na
*Thalassiosira pseudonana*	Na	Na	Na	Na
*Volvox carteri*	Na	Na	Na	Na

Na, not present or not identifiable; numbers indicate GenBank DNA accession numbers.

**Table 6:** Distribution of ATG proteins and domains in microalgae and *M. brevicollis*: ATG proteins exclusively involved in CVT.

	ATG11 (NP_015374.1) (APG11+Cenp-F_leu_zip)	ATG19 (NP_014559.1) (ATG19)/(EHN00336.1) (ATG19)	ATG34 (NP_014558.1) (ATG19)
*Chlorella* sp. NC64A (*Chlorella variabilis*)	Na	Na	Na
*Chlamydomonas reinhardtii*	Na	Na	Na
*Micromonas pusilla* CCMP1545	Na	Na	Na
*Micromonas sp.* RCC299	Na	Na	Na
*Monosiga brevicollis*	Na	Na	Na
*Ostreococcus lucimarinus*	Na	Na	Na
*Ostreococcus tauri*	Na	Na	Na
*Phaeodactylum tricornutum*	XP_002186517.1(ATG11+APG17)	Na	Na
*Thalassiosira pseudonana*	XP_002289246.1(ATG11+Histidinol_dh)	Na	Na
*Volvox carteri*	Na	Na	Na

Na, not present or not identifiable; numbers indicate GenBank DNA accession numbers.

**Table 7:** Distribution of ATG proteins and domains in microalgae and *M. brevicollis*: ATG proteins exclusively involved in pexophagy.

	ATG11 (NP_015374.1) (APG11+Cenp-F_leu_zip)	ATG17 (NP_013527.1) (APG17)	ATG25 (Q6JUT9) (ADIP)	ATG26 (NP_013290.1) (PH+GRAM+Glyco_transf_28+UDPGT)	ATG28 (AAW31632.1) (Na)/(ABO31323.1) (Na)/XP_002491513.1(Na)	ATG30 (XP_002493096.1) (Na)/(BAL15150.1)	PEX3 (Q01497.1) (Peroxin-3)/(NP_010616.1) (Peroxin-3)	PEX14 (BAK41865.1) (PEX14-N)/NP_011362.1 (PEX14-N+PRK00409)
*Chlorella* sp. NC64A (*Chlorella variabilis*)	Na	EFN58270.1 (APG17+ubiquitin+DELLA)	Na	EFN55096.1(UDPGT+Glyco_transf_28) EFN58002.1(GRAM) EFN55594.1(UDPGT) EFN57081.1(Glyco_transf_28) EFN55592.1(UDPGT) EFN58760.1(UDPGT)	Na	Na	EFN54221.1 (Peroxin-3)	EFN56914.1 (Pex14_N)
*Chlamydomonas reinhardtii*	Na	Na	Na	XP_001691133.1 (Glyco_transf_28) XP_001701057.1(UDPGT) XP_001701484.1(GRAM)	Na	Na	Na	Na
*Micromonas pusilla*	Na	XP_003062461.1(APG17+Nucleopla smin+TMF_DNA_bd)	Na	XP_003059429.1(GRAM) XP_003063285.1(GRAM)	Na	Na	Na	XP_003063040.1 (Pex14_N)
*Micromonas sp.* RCC299	Na	XP_002506097.1 (APG17+ubiquitin)	Na	XP_00250 0840.1(GRAM)	Na	Na	Na	XP_002508753.1 (Pex14_N)
*Monosiga brevicollis*	Na	Na	Na	XP_001746818.1(GRAM) XP_001746751.1(UDPGT)	Na	Na	Na	Na
*Ostreococcus lucimarinus*	Na	Na	Na	XP_001421559.1(UDPGT) XP_001421559.1 (UDPGT+Glyco_transf_28) XP_001418405.1(GRAM)	Na	Na	Na	Na
*Ostreococcus tauri*	Na	Na	Na	XP_003079742.1(GRAM) XP_003083497.1(Glyco_tran_28_C)	Na	Na	XP_003083403.1 (Peroxin-3)	XP_003074580.1 (Pex14_N)
*Phaeodactylum tricornutum*	XP_002186517.1(ATG11+APG17)	XP_002186517.1 (APG17+ATG11)	Na	XP_002182817.1 (Glyco_transf_28+UDPGT) XP_002185859.1(GRAM)	Na	Na	XP_002185591.1 (Peroxin-3)	Na
*Thalassiosira pseudonana*	XP_002289246.1(ATG11+ Histidinol_dh)	Na	Na	XP_002294273.1(UDPGT) XP_002287301.1(GRAM)	Na	Na	XP_002292876.1 (Peroxin-3)	XP_002289783.1(Pex14_N+PUB)
*Volvox carteri*	Na	Na	Na	XP_002950839.1(GRAM) XP_002956588.1 (Glyco_transf_28)	Na	Na	Na	XP_002951550.1 (Pex14_N)

Na, not present or not identifiable; numbers indicate GenBank DNA accession numbers.

**Table 8:** Distribution of ATG proteins and domains in microalgae and *M. brevicollis*: Other ATG proteins pexophagy.

	ATG15 (NP_009994.2)	ATG17 (NP_013527.1)	ATG20 (NP_010170.1)	ATG22 (NP_009892.1)	ATG27 (NP_012357.2)
	(Lipase_3 )	(APG17)	(PX+Vps5)	(APG22+MFS)	(ATG27)
*Chlorella* sp. NC64A (*Chlorella variabilis*)	EFN56954.1 (Lipase_3)	EFN58270.1 (APG17+ubiquitin+DELLA)	EFN54738.1 (PX+Vps5)	Na	Na
*Chlamydomonas reinhardtii*	XP_001696400.1 (Lipase_3) ADF43176.1 (Lipase_3) ADF43143.1 (Lipase_3)	Na	XP_001695319.1(PX+Vps5) XP_001690659.1(PX+Vps5)	Na	Na
*Micromonas pusilla* CCMP1545	XP_003059563.1(Lipase_3+Acyl_transf_1)	XP_003062461.1 (APG17+Nucleoplasmin+TMF_DNA_bd)	XP_003055859.1(PX+Vps5) XP_003060307.1(PX+Vps5)	XP_003064473.1 (ATG22) XP_003058402.1 (ATG22)	Na
*Micromonas sp.* RCC299	XP_002509028.1(Lipase_3)	XP_002506097.1 (APG17+ubiquitin)	XP_002505309.1(PX+Vps5) XP_002504443.1(PX+Vps5) XP_002500454.1(PX+Vps5)	XP_002503338.1 (ATG22+MFS) XP_002500650.1 (ATG22+X17NfeD)	Na
*Monosiga brevicollis*	XP_001748884.1(Lipase_3)	Na	XP_001745313.1(PX+Vps5) XP_001750730.1(PX+Vps5)	Na	XP_001748990.1(ATG27) XP_001748431.1(ATG27+Frag1+WSC+Somatomedin_B+Kelch_1) XP_001748596.1(ATG27)
*Ostreococcus lucimarinus*	XP_001417482.1(Lipase_3)	Na	XP_001416534.1(PX+Vps5) XP_001421495.1(PX+Vps5) XP_001417891.1(PX+Vps5) XP_001420137.1(PX+Vps5)	Na	Na
*Ostreococcus tauri*	XP_003082612.1(Lipase_3+Tubulin)	Na	XP_003077939.1(PX+Vps5) XP_003083347.1(PX+Vps5) XP_003081616.1(PX+Vps5) XP_003079106.1(PX+Vps5)	Na	Na
*Phaeodactylum tricornutum*	XP_002186305.1(Lipase_3)	XP_002186517.1(APG17+ATG11)	XP_002179688.1(PX+Vps5)	XP_002181202.1 (ATG22+MFS) XP_002177650.1(ATG22+MFS) XP_002181320.1(ATG22+MFS)	Na
*Thalassiosira pseudonana*	XP_002295280.1(Lipase_3) XP_002287497.1(Lipase_3)	Na	XP_002291461.1(PX+Vps5)XP_002291311.1(PX+Vps5)	XP_002293574.1(ATG22) XP_002291816.1(ATG22+DUF1440)	Na
*Volvox carteri*	XP_002945633.1(Lipase_3)	Na	XP_002955830.1(PX+Vps5) XP_002958583.1(PX+Vps5)	Na	Na

Na, not present or not identifiable; numbers indicate GenBank DNA accession numbers.

**Table 9:** Distribution of ATG proteins and domains in microalgae and *M. brevicollis*: Non-yeast ATG proteins.

	FIP200(Q9ESK9.3) (ATG11+DUF3584+ SMC_prok_B)	DRAM(AAH13773.4)(Frag1) (NP_001015869.1) (Frag1)	ATG101(NP_001092143.1) (DUF1649)	UVRAG AAF53277.1(ATG14) NP_003360.2(ATG14+C2)
*Chlorella* sp. NC64A (*Chlorella variabilis*)	Na	Na	EFN56906.1(DUF1649)	EFN52878(ATG14+NIF) EFN51481(ATG14)
*Chlamydomonas reinhardtii*	Na	Na	XP_001700159.1(DUF1649)	XP_001693556(ATG14)
*Micromonas pusilla* CCMP1545	Na	XP_003061508(Frag1)	XP_003057605.1(DFU1649)	XP_003064431(ATG14)
*Micromonas sp.* RCC299	Na	XP_002501355.1(Frag1) XP_002509248.1(Frag1)	XP_002501928.1(DUF1649)	XP_002502757(ATG14+BAR)
*Monosiga brevicollis*	Na	XP_001744476.1(Frag1) XP_001748431.1(Frag1+ATG27+WSC+Somatomedin_B+Kelch_1+Kelch_6 ) XP_001747358.1(Frag1+SPX) XP_001746889.1(Frag1+SPX)	Na	XP_001745375(ATG14+FmrO+GAS)
*Ostreococcus lucimarinus*	Na	XP_001416868.1(Frag1)	XP_001421448.1(DUF1649) XP_001421417.1(DUF1649)	XP_001422247.1(ATG14) XP_001421563.1(ATG14)
*Ostreococcus tauri*	Na	XP_003078016.1(Frag1)	Na	XP_003084186(ATG14) XP_003083512(ATG14)
*Phaeodactylum tricornutum*	XP_002186517.1(ATG11+APG17)	Na	Na	XP_002177421 (ATG14) XP_002176147 (ATG14) XP_002185098(ATG14)
*Thalassiosira pseudonana*	XP_002289246.1(ATG11+Histidinol_dh)	Na	XP_002287468.1(DUF1649)	XP_002294494.1(ATG14) XP_002288406(ATG14)
*Volvox carteri*	Na	Na	XP_002951085.1(DUF1649)	XP_002948890(ATG14) XP_002953012(ATG14)

### 1. ATG proteins involved in ATG9-Cycling system (ATG1, ATG2, ATG9, ATG13, ATG18, ATG23, and ATG27)

**Figure 1:**
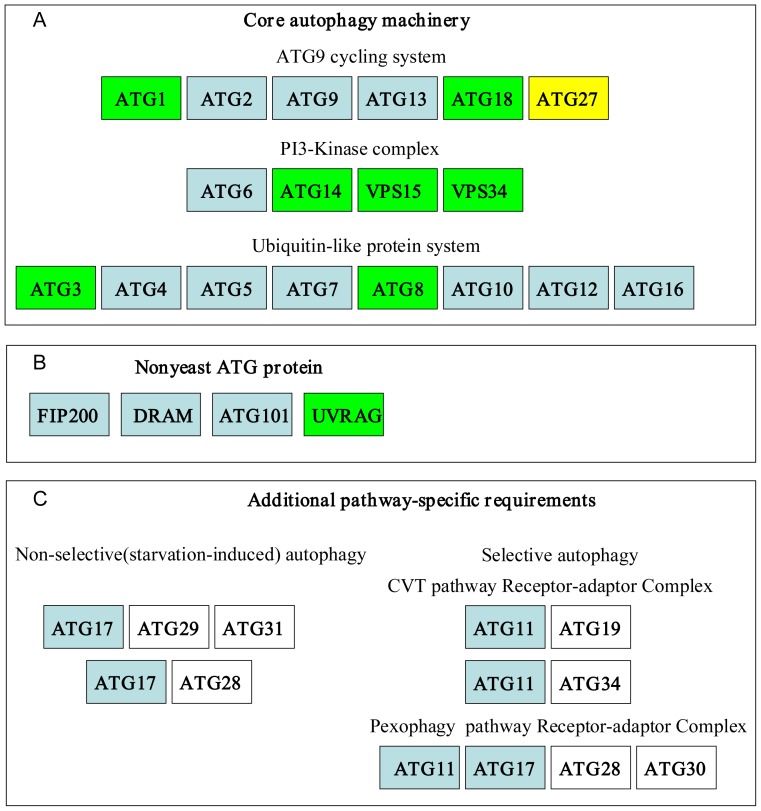
Distribution of autophagy components in microalgae and choanoflagellate. Blank box: no homologues or putative orthologs could be found in any of the seven microalgae genomes; green box: predicted orthologs were detected in each of the genomes studied; blue box: putative orthologs were detected in some algal genomes; yellow box: homologues could only be found in *Monosiga brevicollis*. A: Distribution of putative ATG proteins in “core autophagic machinery”. B: The existence of homologues of non-yeast ATG proteins. C: The existence of the orthologs of additional pathway-specific requirement.

As indicated in Table 2 and Figure 1A, several core proteins in the ATG9-cycling system, including ATG1, ATG2, ATG9, and ATG13, are conserved in most of the species studied. In baker's yeast, cycling of ATG9 between PAS (pre-autophagosomal structure) and non-PAS requires the ATG1 kinase complex (including ATG1 and a regulatory subunit ATG13), ATG2 and ATG18 (two interacting peripheral membrane proteins). A single ATG9 homologue is present in each of the microalgal genomes. However, ATG23 and ATG27 are missing in microalgae. By contrast, *M. brevicollis* maintains three putative ATG27 proteins, which are usually absent in plants and animals. The presence of multiple members of the WD40 family in microalgae, which are equally similar to ScATG18, precludes proper identification of ATG18. Besides, several additional domains identified in ATG1 imply that ATG1 is a multifunctional protein and similar conclusions were obtained from yeast to humans [34]. To our surprise, several core proteins involved in ATG9-cycling system seem to be missing in *C. reinhardtii*, including ATG2, ATG9, and ATG13. These findings are not consistent with the fact that deletion of ATG9 leads to complete abrogation of autophagy, while *atg23 Δ* or *atg27 Δ* strains can perform autophagy at a low level in *S. cerevisiae* [13], [35], [36].

### 2. ATG proteins involved in PI3K complex (ATG6/VPS30, ATG14, VPS15, VPS34 and VPS38)

ATG proteins in the PI3K complex, including VPS34, VPS15, ATG6 and ATG14, are almost fully conserved in microalgae (see Table 3 and Figure 1A). VPS34, the only class III phosphatidylinositol 3-kinase, participates in multiple membrane trafficking events in macroautophagy, Cvt pathway and pexophagy [37]. ScVPS34 contains three domains (including PI3Kc_III, PI3Ka_III, and C2_PI3K_class_III), whereas in several algal orthologs of VPS34, only one or two related domains could be identified, which suggests that the function of VPS34 in yeast may be compensated by two or three proteins in microalgae. For instance, EFN55063.1 of *C. variabilis* maintains PI3Kc_like and FATC domains, while XP_002184171.1 from *Phaeodactylum tricornutum* exclusively maintains a PI3Kc- like domain. Interestingly, although orthologs of ScATG14 required for PI3K complex I were identified in microalgae, an ATG14-like protein was missing in some metazoans (Table 3). This result suggests that the molecular mechanisms of the PI3K complex require species-specific proteins in plants and animals.

### 3. ATG proteins involved in Ubiquitin-Like Protein Conjugation Systems (ATG3, ATG4, ATG5, ATG7, ATG8, ATG10, ATG12, and ATG16)

**Figure 2:**
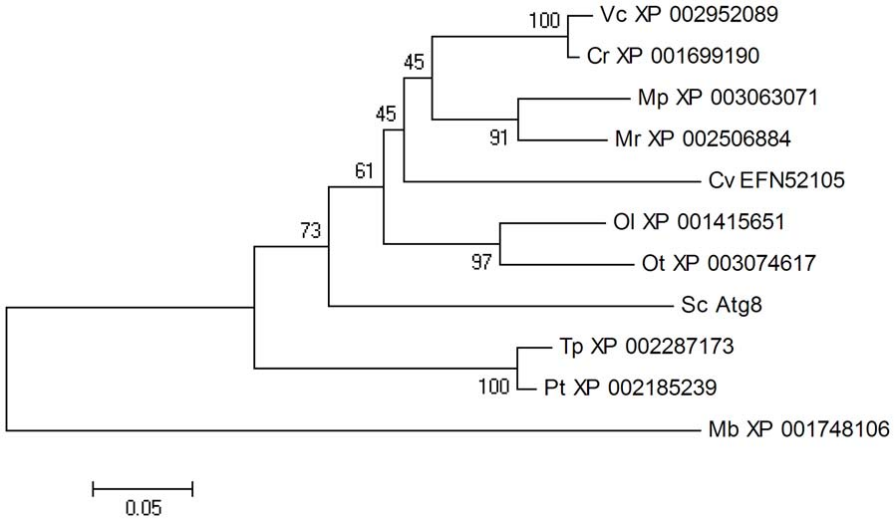
Phylogenetic tree of ATG8 proteins. ATG8 from *M. brevicollis* is used as outgroups. The phylogenetic tree was constructed as described in Methods section (1000 bootstrap replicates). Protein accession numbers and the strain names are as in Table 1 and Table 4, respectively.

Two ubiquitin-like protein conjugation systems are involved in selective and nonselective autophagy, including ATG8-PE (phosphatidyl ethanolamine) and ATG5-ATG12. The first ubiquitin-like protein conjugation complex, composed of ATG3, ATG4, ATG7 and ATG8, is highly conserved in microalgae, except for ATG4 in *M. pusilla* and ATG7 in *P. tricornutum* (see Table 4 and Figure 1A). The phylogenetic tree of ATG8 coincides well with the phylogenies based on 16 s rRNA (Figure 2). As for the second complex, only the green algae *Chlorella* and *Chlamydomonas* contain the entire set of the core proteins. It appears that several algal species including *V. carteri*, *P. tricornutum*, *Micromonas* sp. and *T. pseudonana* lack genes involved in ATG5-12 conjugation. As expected, searches for ATG3 and ATG10 homologs resulted in identical protein sequences, since the region around cysteine234 of ATG3 shows partial homology to the corresponding region surrounding cysteine133 of ATG10. ATG16-like proteins can be identified in certain microalgae, all of which contain mammalian-style multiple WD40 repeats and other additional domains in the extended C-termini relative to their fungal counterparts.

### ATG proteins exclusively involved in non-selective or selective autophagy

**Figure 3:**
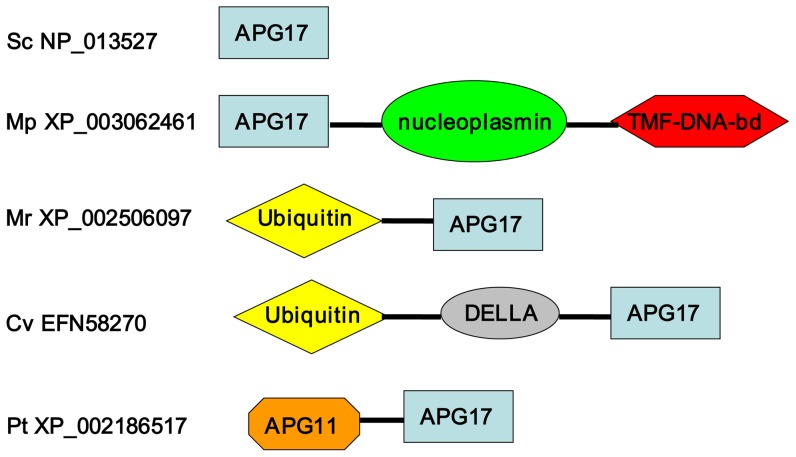
Domain organization of putative ATG17 proteins. Species and sequences are as in Table 1 and Table 7. Fused domains that form a single polypeptide chain are connected by a horizontal line. Figures are not drawn to scale.

As indicated in Table 5 and Figure 1C, ATG28 (in *P. pastoris*) or ATG29-ATG31 (in *S. cerevisiae*), which are exclusively involved in non-selective (starvation-induced) autophagy, are absent in microalgae. On the other hand, ATG17-like proteins are identified in certain species, including *C. variabilis*, *Micromonas* sp. and *P. tricornutum.* Most of the putative ATG17 proteins contain several additional domains, including Ubiquitin and DELLA (Figure 3). It is worth noting that a *P. tricornutum* protein (XP_002186517.1) contains both ATG11 and APG17 domains.

In microalgae, putative ATG proteins belonging to the receptor-adaptor complex in the Cvt pathway, pexophagy and mitophagy are absent (Figure 1C). Orthologs of the Cvt-specific receptor protein ATG19/ATG34 and their adaptor ScATG11 are also uncharacterized, aside from ATG 11 in *P. tricornutum* and *T. pseudonana* (see Table 6). Furthermore, ATG25, ATG28 and ATG30, which are specifically required for *P. pastoris* pexophagy, are not identified. Peroxisomal membrane proteins PEX3 and PEX14 are identified in *C. variabilis*, *O. tauri* and *T. pseudonana* (Table 7). In addition, the newly-discovered mitophagy receptor ATG32 in *S. cerevisiae* is absent in algal genomes. Other ATG proteins responsible for mitophagy, such as ATG33, also cannot be identified (data not shown).

### Identification of non-yeast ATG proteins in microalgae and choanoflagellate

As previously mentioned, we scanned microalgae genomes to identify Sc ATG or Pp ATG homologs. However, several proteins are specifically important for autophagy in mammals, including UVRAG (for activating VPS34), DRAM (Damage-Regulated Autophagy Modulator, like TOR), FIP200 and ATG101 (mediating mTOR signaling) [23]–[27]. Therefore, we also tried to identify their orthologs in microalgae and choanoflagellate genomes (Table 9, Figure 1B). Orthologs of UVRAG are found in all microalgae genomes. On the other hand, DRAM-like proteins are absent from green algae *Chlorella* and *Chlamydomonas*. In addition, Both FIP200 and ATG101 are present in *T. Pseudonana* even though such is not always the case in *Caenorhabditis elegans* and *Drosophila melanogaster* (data not shown). Interestingly, some mammalian ATG proteins and yeast ATG proteins contain similar domains. For instance, the ATG14 domain was discovered in UVRAG and ScATG14, while the ATG11 domain was detected in FIP200 and ScATG11.

### Structure and conserved residues of putative ATGs

**Figure 4:**
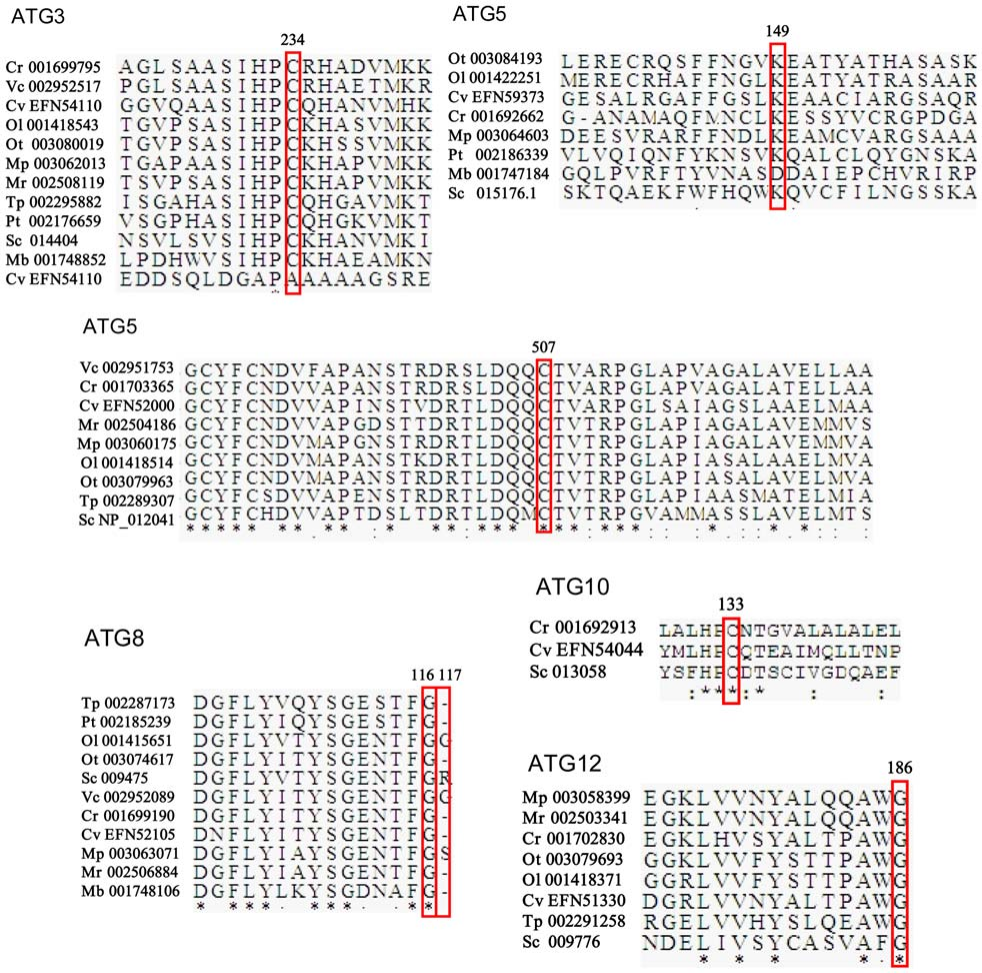
Partial sequence alignment of ATG proteins maintaining conserved catalytic and binding sites. The abbreviations of species are given in Table 1 and the sequence accession numbers are given in Table 4. The asterisks indicate similar residues. Residues in the catalytic and binding sites are in red boxes and the numbers indicate the positions in *S.cerevisiae* (Sc) ATG proteins.

Overall, there are few additional domains present within most of the ATG-like proteins identified in algae. In comparison, in ATG1, ATG17 and DRAM, multiple additional domains were identified (Table 2 and Figure 3). Several crucial residues, conserved in the catalytic and binding sites of ATG proteins in yeast, plants and animals, were also detected in microalgae. For instance, the Cys507 residue of ATG7 (which activates ATG8 and ATG12) is conserved in microalgae (Figure 4). The Cys234 residue in ATG3 (which activates ATG8) and Cys133 of ATG10 (which activates ATG12) are conserved except for EFN54110 in *C. variabilis*. The residues responsible for the formation of the conjugation bond between ATG5 (Lys149) and ATG12 (Gly186) are conserved in all of the algal proteins. The C-terminal cleavage site of ATG8 in baker's yeast, Arg117, could not be identified in most microalgae, but the exposed Gly116 residues are conserved (Figure 4).

### mRNA expression analysis of selected autophagic genes in *Chlamydomonas* and *Chlorella*

**Figure 5:**
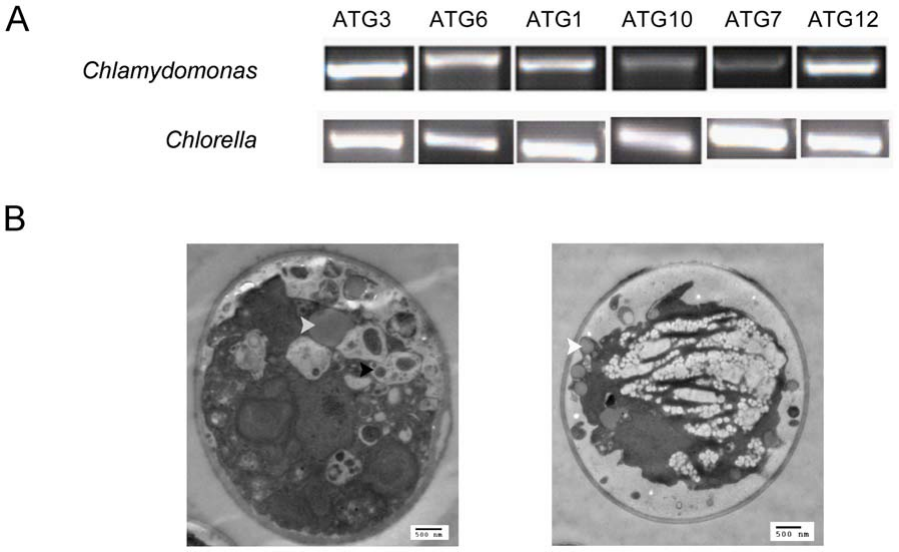
Expression and functional verification of autophagy genes in *Chlamydomonas* and *Chlorella*. A: RT-PCR analysis of selected proteins in *Chlamydomonas* and *Chlorella* to confirm the sequences obtained from in silico research. Total RNA samples were collected during autophagy as described in material and methods section. Data are representative of three independent experiments. B: 3-MA (PI3K pathway inhibitor) blocks the formation of autophagic vacuole in *Chlorella*. Left: Autotrophically grown *Chlorella* cell resuspended in the heterotrophic medium and sampled after 6 h. Autophagic vacuoles disperse throughout the cytoplasm. Right: Autotrophically grown *Chlorella* cell resuspended in the heterotrophic medium for 6 h with 3-MA. There are no obvious autophagic vacuoles. The bar represents 500 nm. White arrowhead: lipid body; black arrowhead: autophagic vacuole. Data represent three independent experiments (A total of 208 control cells/201 test cells were analyzed).

As described above, the green algae *Chlorella* and *Chlamydomonas* contain relatively complete sets of “core autophagy machinery” within their genomes. Therefore, we chose putative ATGs in *Chlorella* and *Chlamydomonas* to confirm if the sequences gained from in-silico research are actually expressed. Total RNA samples were collected during autophagy as described in the material and methods section and RT-PCR analysis of ATG genes was performed. To minimize our work while obtaining more information, we tested ATG1 in the ATG9 cycling system, ATG6 in the PI3K complex, and ATG3, ATG7, ATG10 and ATG12 in the ubiquitin-like protein conjugation systems Ι and Π. The results showed that all the genes tested are expressed during autophagy in *Chlorella* and *Chlamydomonas* (see Figure 5A).

In addition, the expression levels of tested genes during early and late stages of autophagy were also determined (Figure S1). As mentioned in introduction, autophagy can be divided into two stages. Expression levels of tested genes in *Chlorella* cells which were autotrophically-heterotrophically cultured for 2 h/4 h (early stage, before the fusion of autophagosome and vacuole at 6–7 h) and 8 h/10 h (late stage, after the fusion of autophagosome and vacuole at 6–7 h) were tested. The results indicated that the transcription of ATG genes remains constant during autophagy.

### Inhibition of PI3K blocks the formation of autophagic vacuole in *Chlorella*

As mentioned in the introduction, the molecular evidence for autophagic pathways in *C. reinhardtii* has already been reported [29], [38]. Therefore, to extend the results from in-silico and mRNA analysis, we tested the presence and function of the PI3K pathway in *Chlorella*. 3-MA (3-Methyladenine) is a specific inhibitor of PI3K, which can suppress the formation of autophagosomes or autophagic vacuoles (AV). The autophagic vacuoles are defined as membrane-bound vacuoles that contain fragments of cellular components destined for destruction, including mitochondria, endoplasmic reticulum or chloroplasts. The presence of electron- microscopically visible AV is regarded as evidence for autophagy in both plants and *C. reinhardtii*.

We examined 208 control and 201 test cells by electron microscopy (EM) which were switched from autotrophic to heterotrophic growth for 6 h to induce autophagy in three independent experiments. Shown in Fig. 5B is one of the typical cell, in which several autophagic vacuoles disperse throughout the cytoplasm. Among the cells we examined, 168 control cells (81%) and 157 test cells (78%) present similar structure as in Figure 5B. Significantly, when the cells were treated with 3-MA at the same time, no autophagic vacuoles were observed (see Figure 5B), suggesting the formation of these vacuoles depends on autophagy.

## Discussion

In the past five years, due to increased energy consumption and the impact of carbon dioxide emissions on the environment, renewable bioenergy has grown into a research hotspot. A lot of studies have focused on microalgae, which can be used for the production of biodiesel [2]. Recently, we found that an autophagy-like mechanism plays a critical role in the photosynthesis-fermentation conversion and the production of lipids in *Chlorella* (Jiang Q. et. al., paper in preparation), which leads us to perform a complete survey of autophagy machineries in microalgae.

So far, several surveys on autophagy genes have been reported in yeast [20], [21]. Furthermore, based on the ATGs identified in yeast, in silico identifications of ATG genes in filamentous fungi, trypanosomatids, and ascidian *Ciona intestinalis* genomes were performed, which have greatly accelerated our understanding of autophagic pathways in animals and fungi, despite that the function of most ATG genes remains unclear in these studies [22], [39]–[42]. Likewise, the identification of ATG genes in microalgae genomes, especially in those oleic species will be important to understand the molecular mechanism of oil accumulation. Furthermore previous in silico studies of autophagy genes were performed according to the sequential steps of autophagy in yeast and could not differentiate between the core genes conserved from yeast to human and the yeast-specific genes. In this study, the identification of the “core molecular machinery” in microalgae would eliminate the interference of yeast-specific proteins and yield a more precise view of the hereditary conservation and modification of autophagic pathways.

In most cases, comparative genome analyses of autophagy proteins favor the use of ATG genes from *S. cerevisiae* as queries [22], [39]–[42]. However, studies have confirmed that some putative ATG proteins can be identified exclusively using sequences from an organism evolutionarily distinct from yeast [41]. Therefore, in this study, besides *S. cerevisiae*, we also chose ATG proteins from *P. pastoris*, filamentous fungus *Penicillium chrysogenum* and even human as queries. The occurrence of orthologs of mammalian-specific/derived ATG proteins in microalgae (DRAM, ATG101 and UVRAG) and the discovery of common domains in mammalian ATG and yeast ATG (Table 9, Figure 1B) suggests that the pathways and proteins of autophagy are more conserved than previously expected.

### Conservation and the loss of autophagy subtypes in microalgae

The molecular machinery in microalgae is conserved but more complicated than expected (Table 2 to Table 9 and Figure 1A). The green alga *Chlorella* contains the entire set of the core autophagic machinery. In *C. reinhardtii*, however, we could not identify putative orthologs of most ATG genes involved in the ATG9-cycling system (Table 2). In addition, other species seem to lack the second ubiquitin-like protein conjugation complex (Table 4).

In *P. pastoris*, the peroxisome receptor ATG30 interacts with peroxisomes via PEX3 and PEX14, and with autophagy machinery via ATG11 and ATG17 [43]. Thus, the protein (Accession number: XP_002186517) maintaining ATG11 and APG17 domains and the identification of PEX3 and PEX14 imply the presence of the pexophagy mechanism and the modification of receptor proteins in microalgae (Figure 3).

Aside from pexophagy, proteins involved in several subtypes of autophagy were not present (Tables 5, 6, 7 and Figure 1C). For example, the Cvt-specific receptor protein ScATG19/ScATG34 and the mitophagy receptor ScATG32 seem absent in microalgae, which may imply the loss of Cvt and mitophagy pathways or major modifications of the receptors (Table 6 and Figure 1C). Actually, experimental evidence has demonstrated that Cvt is a fungi-specific pathway and some of the proteins involved are lineage-specific duplications in yeast and filamentous fungi [44], [45]. Moreover, the absence of proteins in non-selective autophagy (including macroautophagy and microautophagy) may demonstrate that these proteins are indeed lineage-specific (Table 5 and Figure 1C).

### Expression and functional verification of ATG genes

RT-PCR analysis of putative ATGs in *Chlamydomonas* and *Chlorella* have confirmed that the putative genes identified are actually expressed (Figure 5A). In addition, since sequence similarity and conservation of function are not inevitably connected, experimental studies are required to assure the results obtained from in silico and mRNA analysis. The finding that 3-MA is able to inhibit the formation of autophagy vacuole strongly support the presence of autophagy in *Chlorella* (Figure 5B).

Besides, the induction of autophagy and the functions of several autophagy related genes in *Chlamydomonas* have been experimentally studied recently. For example, CrATG8 (ATG8 protein from *Chlamydomonas*) was proved to be functionally conserved [29]. Like yeast ATG8, CrATG8 is cleaved at the conserved glycine (carboxyl-terminal) and associated with membranes during autophagy induced by different stresses, including nutrient limitation, oxidative stress, and the accumulation of misfolded proteins in the endoplasmic reticulum [29].

### *Chlorella*: a potential model organism to study autophagy

Studies on autophagy in microalgae have only been carried out recently and are mainly using *C. reinhardtii* [38], [46]. Our study suggested that *Chlorella* may represent another potential model system to investigate autophagic pathways in photosynthetic eukaryotes. Firstly, *Chlorella* contains the entire set of the core autophagy machinery (Table 2 to Table 9). RT-PCR analysis verified that all the genes tested are expressed during autophagy (Figure 5A). Moreover, a specific inhibitor of PI3K blocks autophagy in *Chlorella* cells, which indicates the presence of a functional PI3K complex (Figure 5B). Secondly, the autophagic pathways in *Chlorella* seem closer to those in multicellular eukaryotes than those in *S. cerevisiae*. For example, the selective Cvt pathway widely observed in yeast seems to be absent in both *Chlorella* and higher eukaryotes; and the cleavage mechanism of ATG8 in *Chlorella* better resembles that of higher eukaryotes than yeast's (Figure 4). Thirdly, studying ATG genes in *Chlorella* is much easier than in higher plants because the genomes of higher plants have undergone large-scale duplications, combined with plenty of gene gain-and-loss events [47]. Multiple ATG paralogs have thus been identified in higher plants. For instance, *A. thaliana* maintains nine AtATG8 paralogs [48], [49], while *Chlorella* has far fewer putative paralogs (Table 4). Finally, the unique molecular modifications in certain subtypes of autophagy will provide an opportunity to investigate the microalgae-specific autophagy subpathways.

In conclusion, our study not only presents an overall view of the putative autophagy proteins in the “core autophagy machinery” and several subpathways in microalgae, but also verifies the expression profiles and function of representative autophagic pathways by RT-PCR analysis and inhibitor treatment. Unexpected observations suggest that the “autophagy core machinery” in *Chlorella* seems to be more conserved than that in other algal species. Thus, we provide novel evidence for the conservation of the core machinery and the modifications or supplementations for adaptation to unique environmental niches in different microalgal species. Genome-wide in silico analysis of ATG proteins in microalgae will promote understanding of the general features of autophagic pathways and benefit the production of algal-derived bioenergy with future commercial applications. Finally, our data suggest that *Chlorella* may represent another potential model organism to investigate autophagy pathways in photosynthetic eukaryotes.

## Materials and Methods

### In silico analysis

Seven species of microalgae, including two green algae (*Chlorella* NC64A/*Chlorella variabilis*, and *Volvox carteri*), two ancient green algae (*Micromonas pusilla* CCMP 1545 and *Micromonas* sp. RCC299) and diatom *Phaeodactylum tricornutum* CCAP1055/1 were analyzed in this study (Table 1). In addition, the marine choanoflagellate *Monosiga brevicollis*, the closest unicellular relative of animals, was also included.

The sequences of the published ATG proteins were used as queries in local Gapped-Blast analyses to identify putative orthologs of proteins involved in autophagy in microalgae. In most cases, ATG sequences of *S. cerevisiae*, *P. pastoris, Penicillium chrysogenum and Homo sapiens* were used as starting queries. ATG genes not present in yeast but crucial for autophagy in mammals were also detected. The E-value was set to 0.001, and the screening of reciprocal Gapped-Blast was conducted according to the standard protocols [50]. Proteins that maintain the crucial autophagy-related domains were identified and added to the query sets for another round of BLASTp searches. The procedure was continued until no new proteins were found. If no ortholog of Sc ATG was identified in a specific microalga, further analyses were performed using ATGs derived from plants and mammals as queries.

The putative ATG proteins with conserved catalytic and binding residues were aligned in Clustal X [51]. Structure analyses of putative ATGs were performed using SMART, CDD and PFAM [52], [53]. Similar strategies have been employed to study apoptosis (Type I PCD) proteins of cyanobacteria in our lab [54]. A tree based on ATG8 was constructed using NJ methods [55], with the reliability testing of each branch by 1000 bootstrap replications. ATG8 orthologs in *Homo sapiens*, *Arabidopsis thaliana* and *M. brevicollis* were used as outgroups to root the tree.

### mRNA isolation and RT-PCR

*Chlamydomonas reinhardtii* wild-type strain was kindly gifted from Dr. Junmin Pan (Tsinghua University). The induction of autophagy by rapamycin in *Chlamydomonas reinhardtii* was described by José et al. [29]. In brief, cells were cultured and synchronized by alternating light and dark (12 h/12 h) cycles. Cells were treated with 500 nM rapamycin for 24 h. The *Chlorella* cells were grown autotrophically for five days before changing to the heterotrophic medium and sampled after 2, 4, 8 and 10 hours as described previously [56]. Total RNAs were isolated using RNeasy Plant Mini kit according to the instructions (QIAGEN). RT-PCR was performed on equal input mRNA and cDNA amounts and monitored by actin control primers. The PCR products were run on 2.5% agarose gels. Sets of forward (F) and reverse (R) primers for each selected gene and the expected sizes of PCR fragments are listed in Table 10.

**Table 10:** Sets of forward and reverse primers designed to amplify ATG genes.

Gene	Forward primer (F)	Reverse primer (R)	PCR fragment size (bp)
ATG3	CGAGGCAGATGATGAGGCT	GGACGCGATGAACTTGAGAA	388
ATG1	AAACTCTGGGTCAACAAAGGG	TTGCTATGGCATCAGGGAAG	427
ATG6	GGAGGAGTCCTTTGTGATGCT	GCTTGATGGTGGCGTTGTT	932
ATG7	GTCGTCGGCTACTTCAGTCCCA	GCCACATGCTGCCTCATTCC	470
ATG10	ACAATGGACCGCAACATCTGA	ATGACGGGCACCTGGTAGG	468
ATG12	CAATGCGAAGCCGAGGAG	CTGCGATATTCAAACCAGACCA	401

### Electron microscopy

Autotrophically grown *Chlorella* cells were resuspended in the heterotrophic medium and sampled after 6 h either with or without 5 mM 3-MA [56]. Samples ware pretreated using standard protocol, including dehydration, mixation, embedding and sectioning [56]. Cells were photographed using a JEM-1230 transmission electron microscope (Hitachi, Japan).

## Supporting Information

Figure S1 **Expression levels of autophagy genes during autophagy in** ***Chlorella*** **.** Total RNA was collected in *Chlorella* cells which were autotrophically-heterotrophically cultured for 2 h/4 h (early stage, before the fusion of autophagosome and vacuole at 6–7 h) and 8 h/10 h (late stage, after the fusion of autophagosome and vacuole at 6–7 h). Actin was used as loading control. Data are representative of three independent experiments.
(TIF)
