# Supplementary figures and images for: Analysis of Autophagy Genes in Microalgae: Chlorella as a Potential Model to Study Mechanism of Autophagy
Source: PLoS One. 2012 Jul 27;7(7):e41826. doi: 10.1371/journal.pone.0041826 (PMC3407069; doi:10.1371/journal.pone.0041826)

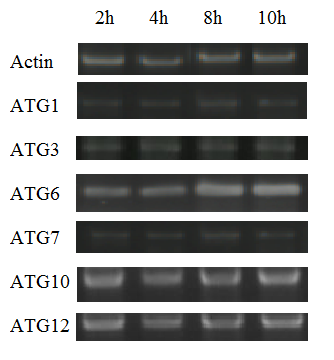

Supplement: Figure S1 — Expression levels of autophagy genes during autophagy in Chlorella . Total RNA was collected in Chlorella cells which were autotrophically-heterotrophically cultured for 2 h/4 h (early stage, before the fusion of autophagosome and vacuole at 6–7 h) and 8 h/10 h (late stage, after the fusion of autophagosome and vacuole at 6–7 h). Actin was used as loading control. Data are representative of three independent experiments. (TIF) [file pone.0041826.s001.tif]
